# A Study on Microstructure and Properties of PHS Fiber Laser Welded Joints Obtained in Air Atmospheres

**DOI:** 10.3390/ma11071135

**Published:** 2018-07-04

**Authors:** Qian Sun, Hong-Shuang Di, Xiao-Nan Wang, Xia-Ming Chen, Xiao-Nan Qi, Jian-Ping Li

**Affiliations:** 1 State Key Laboratory of Rolling and Automation, Northeastern University, Shenyang 110819, China; 1510204@stu.neu.edu.cn (Q.S.); xiaonan_qi@126.com (X.-N.Q.); ljp@mail.neu.edu.cn (J.-P.L.); 2 Shagang School of Iron and Steel, Soochow University, Suzhou 215021, China; chenxiaming@126.com

**Keywords:** laser welding, press-hardened steels, welding atmospheres, Al–Si layer, microstructure and properties

## Abstract

Press-hardened steels (PHS) with a 1.5-mm-thick coated Al–Si layer is welded using an IPG YLS6000 continuous fiber laser in the air atmosphere. An SU5000 scanning electron microscope (SEM) and an Oxford EDS X-Max20 energy spectrometer are used to characterize the microstructure, which consists of delta (δ)-ferrite and lath martensite. It is similar to that of the welding performed in the Ar atmosphere, but the content of δ-ferrite is less. The reason is the formation of Al_2_O_3_ inclusions in the molten pool, which reacts with oxygen from the air ambient and the Al from the molten Al–Si coating of PHS. The oxygen content is measured with an ONH-3000 analyzer. An HV-1000 microhardness tester and DNS-100 universal material test equipment are performed to test the hardness and tensile strength. Similar hardness and strength of welded joints are achieved welding in the air atmosphere compared to that of the Ar atmosphere. Fracture was initialed in the fusion line of overlapping zone and propagated along the interface of two plates and fusion line due to the Al segregation.

## 1. Introduction

With the rapid development of the automotive manufacturing, the safety and energy-saving and emission-reduction have become increasingly prominent. The application of ultra-high strength steels on automotive manufacturing is the key measure to solve these two issues [1]. Press-hardened steel (PHS) is a kind of typical ultra-high strength steels, with a tensile strength of 1500 MPa, and has been widely used in automotive anti-collision beams, front and rear bumpers, A column, B column and the middle passage and other important compounds [2]. In order to ensure the ultra-high strength of PHS, it is heated to 900–950 °C for 5–10 min before hot stamping. Generally, an Al–Si layer is coated on the surface of the steel to prevent surface oxidation during hot stamping [3].

Laser welding has been paid more and more attention to due to its lower heat input, high ratios of penetration depths to weld widths, narrow heat affected zones (HAZs) and small welding deformation. Recently, many researchers have made studies on the joining of PHS using laser welding [4,5,6,7]. Traditional welding theory holds that protective measures must be selected to ensure the performance of welded seam (WS), such as gas-shielded, slag protection and absence of air. In this case, oxidation and nitridation are successfully prevented once air enters the welding pool. In comparison, laser welding has high energy density (~10^6^–10^8^ W/cm^2^), and rapid heating and cooling rates (~10^2^–10^3^ °C/s), short hold time in the welding pool [8,9,10]. The chemical and metallurgical reaction in the welding pool is significantly different from traditional fusion welding (GMAW and GTAW, etc.). Many studies involving the effect of gas atmospheres on microstructures and properties of welded joints were carried out. Bárta and Keskitalo et al. [11,12] used a CO_2_ laser to weld duplex stainless steels, and the difference in microstructures and properties of welded joints with different welding atmospheres was comparatively analyzed. They found that the WS with no welding defects was obtained in N_2_ atmospheres, with a smooth surface. Moreover, a good bending performance was for the WS, where the austenite content was increased from 24% to 56%. Yadaiah et al. [13] studied the depth-to-width ratio and surface morphology of 304 stainless steels welded joints in Ar and air atmospheres using fiber lasers. The results indicated that the WS had a rough surface in the air atmosphere, because the O/N from air induces metallurgical reaction with molten metal in the welding pool. Ar gas compressed the plasma cloud and reduced the shielding effect, so that the depth-to-width ratio approached 29%, more than that of air atmospheres. In addition, the weld width was increased by 40%. For the welding of high strength galvanized steel sheets in pure N_2_ atmospheres, the reaction between the N element and the molten metal occurred in the welding pool to form Fe_4_N nitrides that resulted in a reduction of toughness [14]. In summary, the welding gas atmospheres had a great influence on the microstructure and properties of laser-welded joints, but the studies on the laser welding of PHS in air atmospheres are limited.

Thus, this present work dealt with the laser welding of PHS with the Al–Si layer performed by a fiber laser in the air atmosphere, the microstructure, hardness, inclusions, oxygen (O) content and mechanical properties of welded joints were studied. In addition, the study results with the Ar-atmosphere welding were presented as a comparison in this paper. This study provides an important theoretical basis and a process for laser welding of PHS in the air atmosphere and the following laser welding without removing the Al–Si layer in the oxidizing atmosphere. 

## 2. Experimental Procedures 

### 2.1. Materials

**Figure 1:**
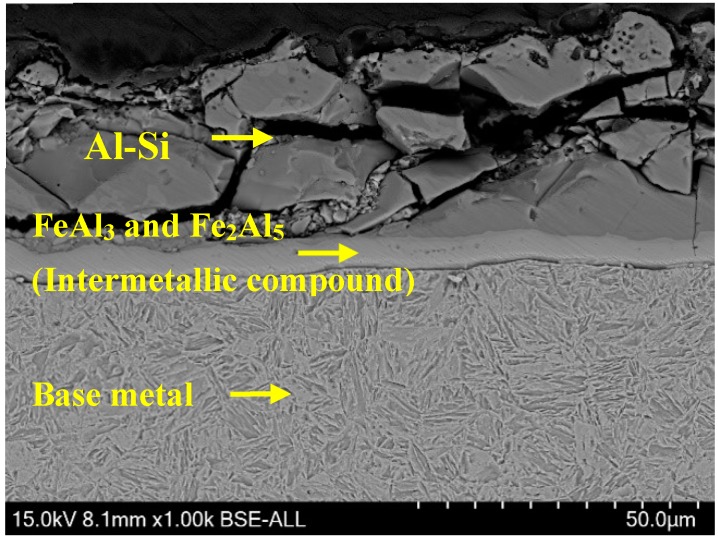
The experimental steel with an Al–Si layer.

The experimental material was 1.5-mm-thick pressed-hardened steel (PHS) with an Al–Si layer, and the microstructure consisted of ferrite (F) and pearlite (P). Table 1 lists the chemical composition of experimental steel. According to previous studies [15], the experimental materials were heated to 950 °C for 5 min in a box resistance furnace, and then quenched to room temperature. The microstructure was lath martensite (LM), as shown as in Figure 1. The surface of PHS had a coating thickness of 40–45 μm, and the inner and the outer were the Al–Si layer and the Al–Fe (FeAl_3_, Fe_2_Al_5_) intermetallic compound (IMC) layer, respectively [16]. 

### 2.2. Experimental Methods

**Figure 2:**
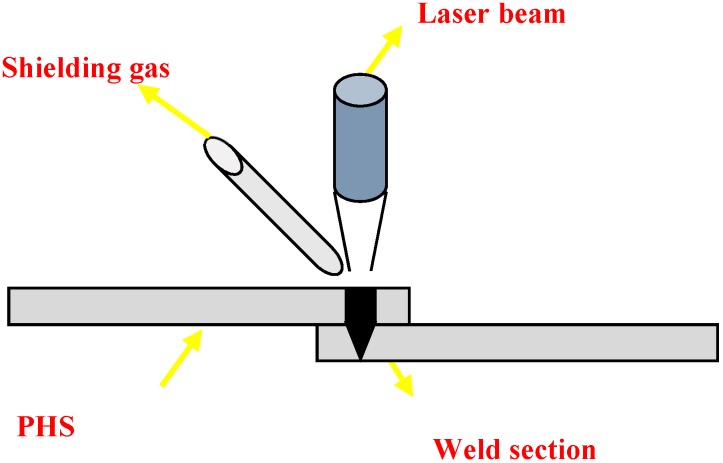
Schematic of laser lap welding.

Welding experiments were carried out using an YLS6000 (IPG, Oxford, MA, USA) continuous laser in two kinds of welding gas atmospheres, with a sample size of 80 × 55 × 1.5 mm (Figure 2). First, the laser welding was performed in the air atmosphere. Second, Pure Ar was selected as the welding shielding gas with a side-blown flow of 15 L/min. Laser power was 4 kW, and the welding speed was 3.00 m/min. The focal length, spot diameter and defocus were 300 mm, 0.4 mm, and 0 mm, respectively.

Samples perpendicular to the WS were polished and then etched with 4% nital. SU5000 scanning electron microscope (SEM, Hitachi, Tokyo, Japan) was used to observe the microstructure of the welded joint, and the element-distribution was analyzed by an EDS X-Max20 energy spectrometer (Oxford, UK). The O content in the WS was measured using an ONH-3000 analyzer (NCS, Beijing, China), with an analysis accuracy of 1 ppm or RSD ≤1%. Line profiles (5 mm) of Vicker microhardness of the welded joint with a step of 0.1 mm were measured using an HV-1000 microhardness tester (Yuzhi, Shanghai, China) at a load of 2.94 N and a loading time of 10 s. The test location was 0.5 mm from the upper surface of the samples and the welding center was the testing center.

**Figure 3:**
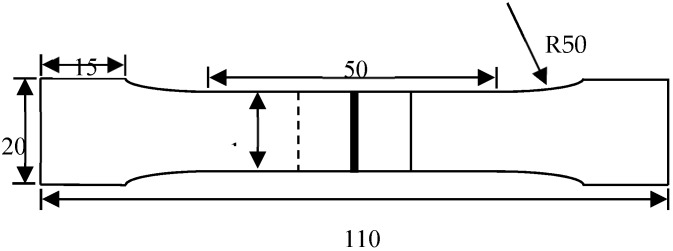
Schematic of tensile shear samples (unit: mm).

According to GB/T 228-2010, a tensile shear test was conducted via the DNS-100 universal material test equipment (Changchun Institute of mechanical research, Changchun, China) at a constant strain rate of 3 mm/min, and the schematic of tensile shear samples was shown in Figure 3. Tensile shear strength of welded joints was calculated by an equation of δ_C_ = *F*/*a·b*, where *F* is shear force in the unit of N; *a* is the actual width of the lapping zone (observed cross section by SEM) in the unit of mm; and *b* is the total length of WS in the unit of mm. Tensile fracture and microstructure of side surface were observed with an SEM. 

## 3. Results and Discussion

### 3.1. Appearance of Welded Seam

**Figure 4:**
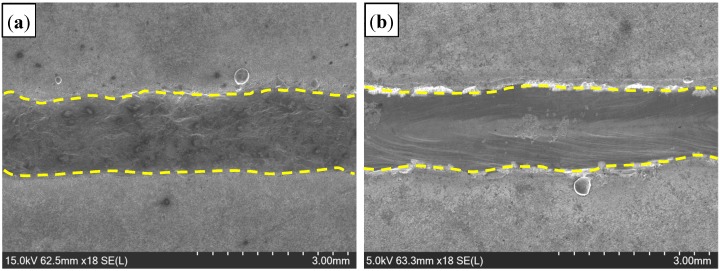
Appearance of welded seam with different welding gas atmospheres: (**a**) air; and (**b**) Ar.

**Figure 5:**
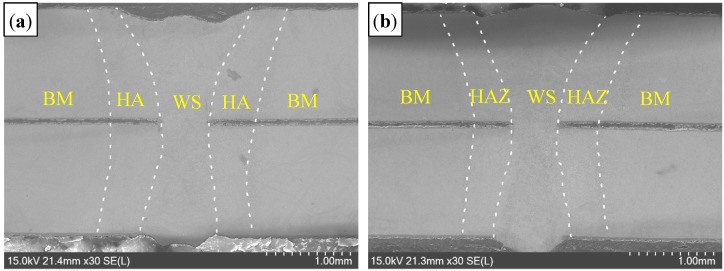
Cross section of welded joints with different welding gas atmospheres: (**a**) air; and (**b**) Ar.

Figure 4 presents the appearance of welded seam (WS). Compared to the welding in the Ar atmosphere, and the WS with a rough surface was obtained in the air atmosphere. Figure 5 presents the overviews of welded joints, consisting of WS, HAZ and base metal (BM). It was evident that the completely weld penetration was obtained in the air atmosphere, with absence of porosity and cracks, etc., which was similar to that in the Ar atmosphere.

For the laser welding in the air atmosphere, the surface oxidation of molten pool easily occurred without supplying shielding gas, resulting in a rough surface for the WS. Traditional welding theory believed that the O content in the WS was significantly increased in the air-atmosphere welding, so that the mechanical properties of welded was deteriorated. However, results obtained with the ONH analyzer showed that the O content was only 95 ppm, which was 1.6 times of that in the Ar atmosphere. Due to the rapid cooling rate and short hold time at high temperature during laser welding [9], the molten metal did not have enough time to dissolve O_2_ that was from the surrounding air ambient. In addition, Gedeon and Eagar [17] suggested that single O atom was dissolved in the molten pool rather than diatomic. Generally, the O_2_ was difficult to induce ionization in this condition due to great ionization energy (13.62 eV), leading to less O content involved during subsequent solidification. 

For fusion welding of filler wire, decomposition and dissolution of gases occurred in the droplet reaction zone [17,18] due to its large specific surface area, high temperature (highest temperature was 2800 °C) and intense reaction. However, the droplet reaction zone was absent during laser welding, resulting in a significant reduction of gas dissolution. Consequently, the laser-induced WS had a satisfactory internal quality in the air atmosphere compared to traditional fusion welding.

### 3.2. Microstructure 

#### 3.2.1. Microstructure of Welded Seam

**Figure 6:**
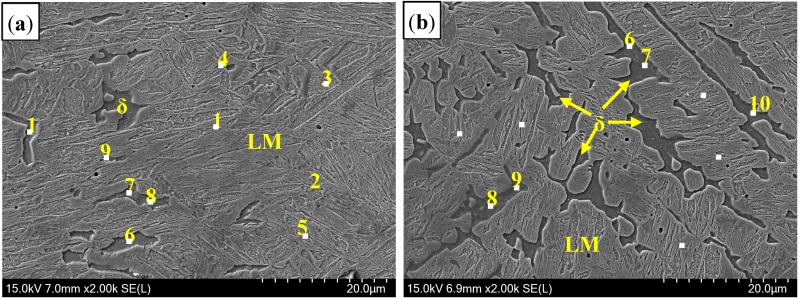
Microstructure of WS with different welding gas atmospheres: (**a**) air atmosphere; and (**b**) Ar atmospheres. Point 1–5 was in martensite, and point 6–10 was in δ-ferrite.

Figure 6 presents the microstructure of welded seam (WS). In the air atmosphere, the microstructure consisted of δ-ferrite and LM [19], similar to that of Ar atmospheres. Delta (δ)-ferrite was formed as individual particles between martensite, which related to the grain wetting behavior during peritectic reaction. 

**Figure 7:**
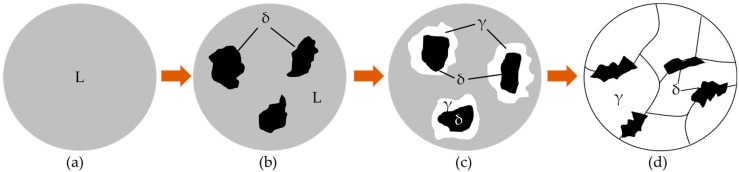
The schematic diagram of the peritectic reaction of experimental steel. (**a**) Liquid phase; (**b**) solid-phase δ-ferrite was separated from liquid phase by crystallization; (**c**) Peritectic reaction occurred: L + δ → γ; (**d**) Peritectic reaction finished and δ-ferrite was retained.

As shown in Figure 7, the welded metals were completely melted in the molten pool by the effect of laser radiation (Figure 7a). During the cooling process, solid-phase δ-ferrite was separated from liquid phase by crystallization (Figure 7b). Then, the two phases induced the peritectic reaction to form the γ-austenite phase (L + δ → γ), where γ phase depended on the nucleation of the δ phase and gradually surrounded it, and finally the liquid metals and the δ-ferrite solid solutions involved in the reaction were separated from γ-austenite (Figure 7c). Thus, the solid/solid interface was found between γ-austenite and δ-ferrite phases, and the δ-ferrite morphology was determined by their grain wetting. Since different crystal structures were for γ-austenite (face-centered cubic) and δ-ferrite (body-centered cubic), the grain boundary energy of both phases was higher than that of the coherent interface or the semi-coherent interface. Based on the studies of Straumal et al. [20], second solid phase formed individual particles along grain boundaries of the matrix solid phase at a high temperature range for the solid/solid interface. At this stage, γ-austenite was incompletely wetted by δ-ferrite, and discontinuous δ-ferrite was retained along the grain boundaries of γ-austenite (Figure 7d).

Generally, δ-ferrite was a high-temperature actor; the content at room temperature was related to alloying elements and the cooling rate. During laser welding, the molten Al–Si layer diffused into the molten pool, so that Al contents increased in WS. Moreover, the Al element was a stronger ferrite stabilizer, which can significantly improve the stability of high-temperature δ-ferrite [21], and it was retained to room temperature during the following solidification. Thus, it was inferred that the presence of Al element was one of the reasons resulting in the formation of δ-ferrite. Yi et al. [22] in their study of δ-TRIP steel found that the high-temperature δ-ferrite cannot be retained to room temperature through the dissolution effect of Al element, unless the Al content was more than 3.0%. Thus, the elemental distributions in δ-ferrite of WS were analyzed, and the results are listed in Table 2. It was found that the average content of Al element in the air atmosphere was 1.70%, less than 3% in the previous study [22]. In comparison, the average Al content in the Ar atmosphere was 2.00%. Further studies showed that the rapid cooling rate (10^2^–10^3^ °C/s) and less hold time at high temperature during laser welding [8,9] were the other reason for the formation of δ-ferrite. In this case, the δ-ferrite with high Al content at high temperature had no adequate time to induce paratactic reaction to form γ-phase, and as a result, retained to room temperature [23]. 

In addition, it was found in Figure 6 that the content of δ-ferrite in the Ar-welding atmosphere was more than that in the air atmosphere, which was due to more O contents diffused into the molten pool to form Al_2_O_3_ inclusions in air welding. The analysis on inclusions will be presented in next section. 

Consequently, the microstructure of WS in air atmospheres consisted of δ-ferrite and LM. High Al contents and the rapid cooling rate were the reasons for the formation of δ-ferrite. Compared to that in the Ar atmosphere, the content of δ-ferrite in air atmospheres was smaller due to more O contents diffused into the molten pool during laser welding. 

#### 3.2.2. Inclusions of Welded Seam

**Figure 8:**
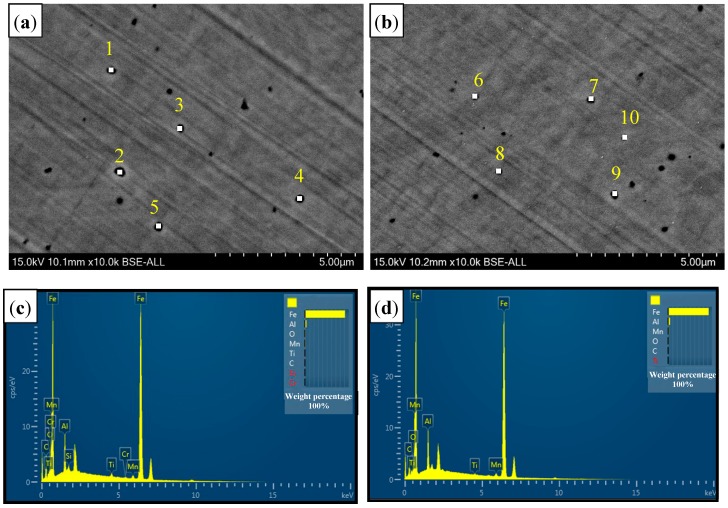
The distribution and EDS analysis of inclusions in WS with different welding gas atmospheres. (**a**) Inclusions distribution in the air atmosphere with typical 5 points (Number 1–5); (**b**) inclusions distribution in the Ar atmosphere with typical 5 points (Number 6–10); (**c**) EDS analysis of the air atmosphere; and (**d**) EDS analysis of the Ar atmosphere.

**Figure 9:**
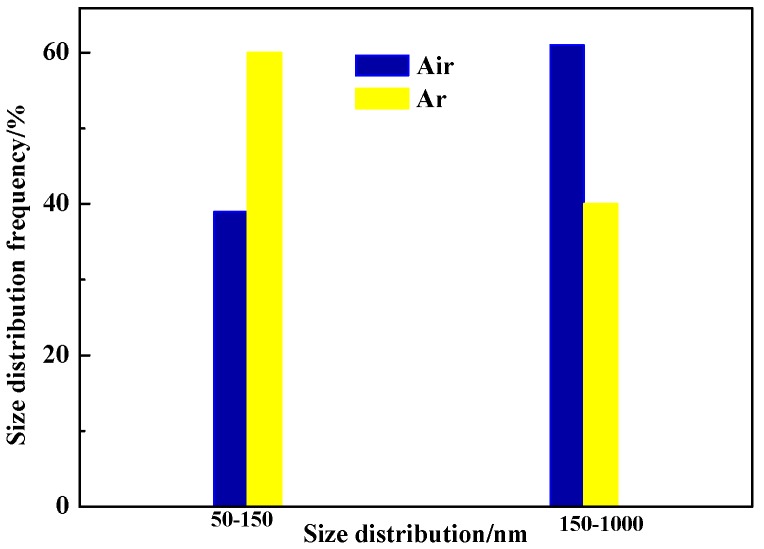
Size-distribution of inclusions with different welding gas atmospheres (unit: nm).

Figure 8 presents the distribution of inclusions in welded seam (WS). EDS analysis results of five typical inclusions were listed in Table 3. The average Al and O contents in air (Al: 3.61%, O: 0.65%) and in Ar atmospheres (Al: 3.16%, O: 0.75%) were observed. Thus, Al_2_O_3_ was inferred as the composition of inclusions. IPP statistical software was used to make a mathematical statistics of the inclusions in WS and 30 random fields were selected; the statistical results are presented in Figure 9. It was showed that the distributional density of inclusions in air atmospheres was 1.10 × 10^5^/mm^2^, which was 1.375 times of that in Ar atmospheres (0.80 × 10^5^/mm^2^), and the inclusions with a diameter of 150–1000 nm approached 61%, which was 1.5 times of Ar atmospheres. 

Generally, most of the O element in the molten pool was preferred to react with the Al element to form Al_2_O_3_ due to great activity compared to SiO_2_ [24,25], and the reaction between Al and O was given in Equation (1):

2[Al] + 3[O] = Al_2_O_3_(s)
*K*’ = *a*^2^_*Al*_·*a*^3^_*O*_/*a*_*Al*2*O*3_ (1)

where *K* is reaction equilibrium constant; *a*_Al_ = *f*_Al_*·*[*%*Al], activity of Al in the molten pool; *a*_O_ = *f*_O_*·*[*%*O], activity of O in the molten pool; *a*_Al_2___O_3__ = 1. Al and O contents were calculated when the reaction reached equilibrium by Equation (1). 

For air-atmosphere welding, the O was ionized and quickly diffused into the molten pool due to the absence of inert gas shielding, leading to an increase of O activity. Compared to Ar atmospheres welding, more Al reacted with O to form Al_2_O_3_, resulting in a great distributional density of inclusions. In addition, the O potential was also improved due to the increase of O content in the molten pool, so that Al_2_O_3_ inclusions had a greater size than that of Ar atmospheres [26]. Thus, the more the O content is, the more the inclusion is and the greater the size is. It also explained the reason that the content of δ-ferrite in air atmospheres was less than that of Ar atmospheres.

#### 3.2.3. Microstructure of Fusion Line

**Figure 10:**
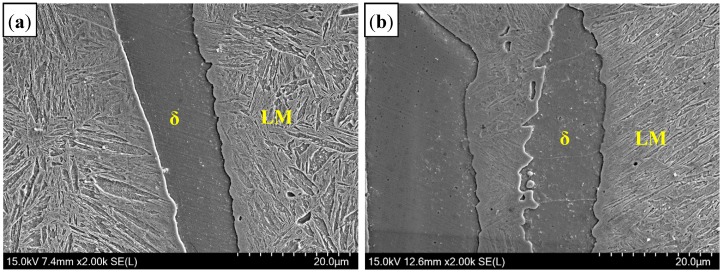
Microstructure of fusion line with different gas atmospheres: (**a**) air; and (**b**) Ar.

**Figure 11:**
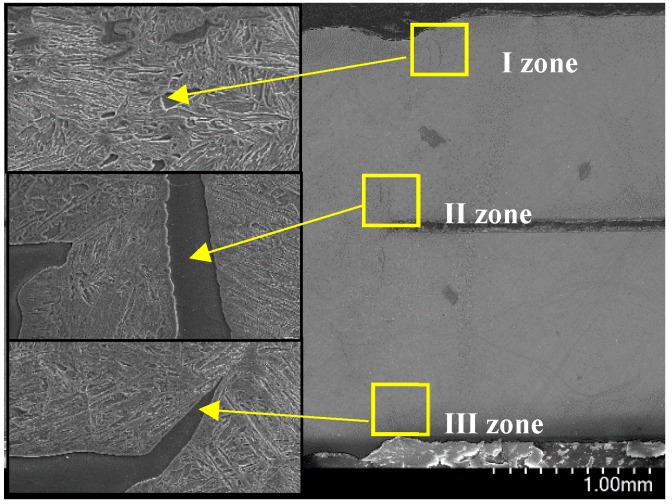
Three zones in fusion line in the air atmosphere.

Figure 10 presents the microstructure of fusion line (FL). Compared to that in the Ar atmosphere, a similar microstructure was observed in the fusion line of the air atmosphere. It consisted of LM and banded δ-ferrite, but the content of δ-ferrite was few. Three zones were selected to measure the Al content in δ-ferrite, as shown as in Figure 11. Based on the EDS analysis results (Table 4), the Al content in δ-ferrite was largest near the overlapping zone (II zone), but that at the bottom was lowest (III zone).

During laser welding, the Al–Si layer coated on the surface of PHS was melted and diffused into the molten pool, so that Al segregated in the fusion line. Previous studies showed that the Al content in δ-ferrite of fusion line on the top surface was greater than that in other locations during PHS tailored welding [5]. At this stage, the Al–Si layer in the overlapping zone was contributed by two-welded surface, so that the Al-segregation was more serious than that on the top surface. In addition, the Al contents in the three zones were smaller than that in the Ar atmosphere; the reason was similar to the analysis of WS.

### 3.3. Hardness and Tensile Shear Strength

**Figure 12:**
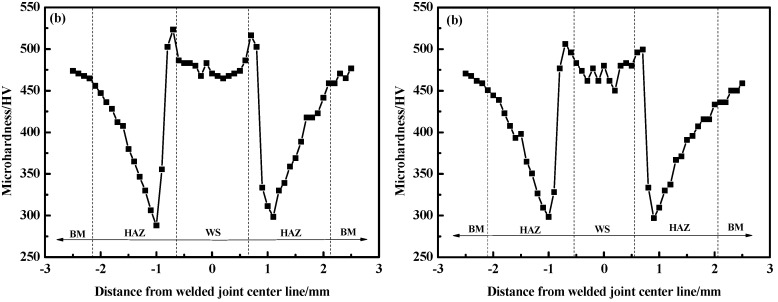
Hardness distribution of welded joints with welding gas atmospheres: (**a**) air; and (**b**) Ar.

Figure 12 presents the hardness distribution of welded joints. In air atmospheres, the average hardness of WS was 458–478 HV, which was higher than base metal (BM) (459 HV) (Figure 12a). The highest hardness value (499–510 HV) was found in the heat affected zone (HAZ) near the side of WS, and the lowest value (290–310 HV) was found near the BM side. 

**Figure 13:**
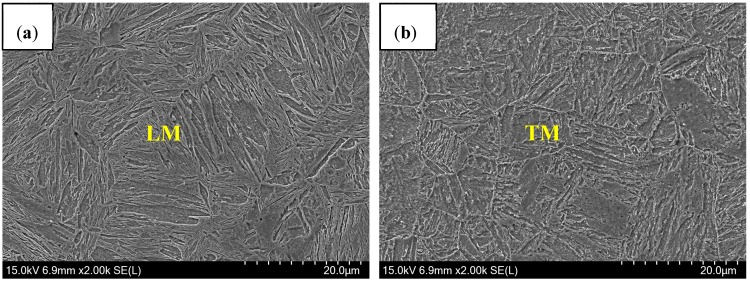
Microstructures of heat affected zone near the WS side (**a**) and near the base metal side (**b**).

Generally, the microstructure was the primary key to determine the hardness [27]. For both welding gas atmospheres, the microstructure of WS was similar only with little difference in the content of δ-ferrite. Thus, the hardness in the air atmosphere had a similar result with that in the Ar atmosphere (Figure 12b). Since welding gas atmospheres only had an influence on the molten metals, the microstructure of HAZ was the same in despite of whether the WS was exposed to Ar or air atmospheres. Figure 13 presents the microstructure of different zones in HAZ. Full martensite with coarse-grain size was observed near the side of WS, resulting in a higher hardness than that near BM. In contrast, the microstructure near the BM side consisted of tempered martensite, leading to a hardness reduction compared to BM. 

**Figure 14:**
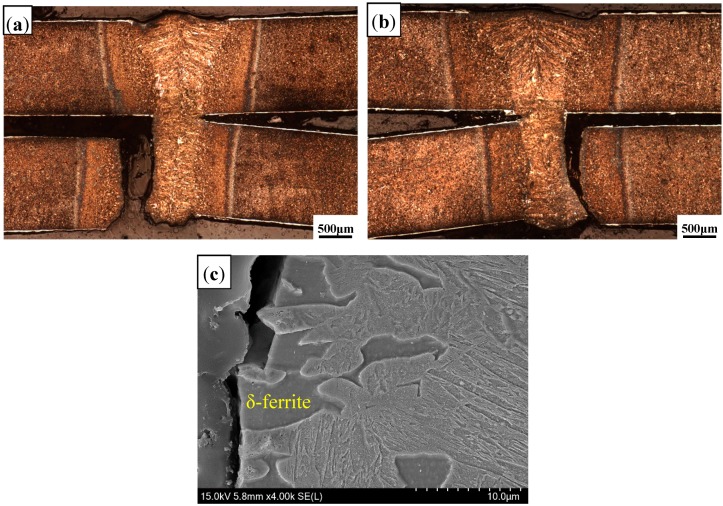
Fracture morphology of cross sections of welded joints in air atmosphere (**a**); and Ar atmosphere (**b**). (**c**) Magnified view of (**a**,**b**).

**Figure 15:**
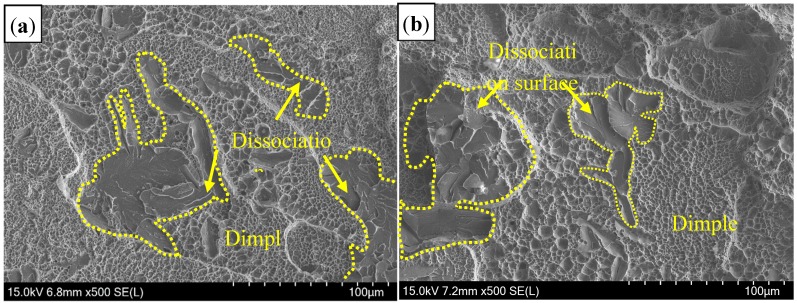
Tensile shear fracture with different welding gas atmospheres: (**a**) air; and (**b**) Ar.

Tensile shear testing results showed that the tensile shear strength of welded joints in air atmospheres was 790 MPa, slightly lower than that of Ar atmospheres (810 MPa). Figure 14 presents the fracture morphology on the cross section of welded joints. It was shown that failure occurred in the fusion line of the overlapping zone for both welding gas atmospheres, and propagated along the interface of two sheets and fusion line (Figure 14a,b). Further observation showed that the fracture initiation was in δ-ferrite (Figure 14c). As shown in Figure 15, the fracture of welded joint was mainly ductile fracture with equiaxed-dimples and accompanied by brittle features for both welding gas conditions.

Compared to Ar atmospheres, the WS in the air atmosphere had a rough appearance and relatively great contents of Al_2_O_3_ inclusions, but the tensile shear strength did not have a significant reduction. The Al_2_O_3_ inclusions with a size of less than 1μm had no influence on the mechanical properties [25]. At this stage, the Al–Si layer on the interface was formed by two surfaces of welded plates. During laser welding, the molten Al–Si layer diffused into the welding pool, so that Al segregated in the fusion line, and it was serious in the overlapping zone. As mentioned, δ-ferrite was formed in the rich-Al zone during the non-equilibrium solidification process and it was a brittle microstructure. Thus, the fracture was initialed in the fusion line and the brittle fracture zone was observed. 

## 4. Conclusions

Laser lap welding on the PHS coated Al–Si layer with 1.5 mm thick was carried out using a fiber laser in the air atmosphere. The microstructure, hardness, and inclusions, O contents and mechanical properties of WS were studied. The conclusions were as follows.

(1) Completely welded penetration was obtained with the absence of defects. The microstructure of WS and fusion line consisted of LM and δ-ferrite due to the molten Al–Si layer. The content of δ-ferrite in the air atmosphere was less than that in the Ar atmosphere due to the reaction between the O and Al elements in the molten pool. γ-austenite was incompletely wetted by δ-ferrite during the peritectic reaction.

(2) The content of O element was 95 ppm, which was 1.6 times of that in the Ar atmosphere. A great size was for Al_2_O_3_ inclusions, of which distributional density (1.1 × 10^5^/mm^2^) was 1.375 times of that in the Ar atmosphere (1.1 × 10^5^/mm^2^), as a result of an increase of O activity with the absence of inert gas shielding.

(3) Compared to those in the Ar atmosphere, similar hardness and tensile shear strength of welded joints were achieved welding in the air atmosphere. Fracture was initialed in the fusion line of the overlapping zone and propagated along the interface of two plates and fusion line due to the Al segregation.

(4) The laser-welded joint of the PHS coated Al–Si layer in the air atmosphere had a similar microstructure and properties with that of shielding gas, which was very different from the traditional welding theory.

## Figures and Tables

**Table 1:** Chemical compositions of experimental steel (mass fraction, %).

C	Si	Mn	P	S	N	Al	Ti	B	Cr
0.23	0.27	1.13	0.013	0.001	0.0042	0.037	0.038	0.0025	0.16

**Table 2:** Elemental distribution of WS in the Ar atmosphere (mass fraction, %).

	Ar					Air				
No.	1	2	3	4	5	6	7	8	9	10
Al	1.92	1.98	1.99	2.14	2.00	1.80	1.57	1.62	1.74	1.67
Si	0.31	0.35	0.56	0.47	0.54	0.38	0.30	0.33	0.38	0.36
Fe	96.77	95.14	96.27	94.76	95.29	95.11	95.80	95.68	95.46	95.54
Others	Balance									

**Table 3:** EDS Analysis of inclusions in WS with different welding gas atmospheres (mass fraction, %).

	Ar					Air				
No.	1	2	3	4	5	6	7	8	9	10
Al	3.58	3.91	3.16	2.50	2.64	4.13	3.93	2.86	3.53	3.63
Si	0.36	0.37	0.33	0.41	0.39	0.33	0.34	0.32	0.37	0.40
Fe	91.74	92.12	88.86	94.55	93.96	92.71	93.38	90.08	93.29	94.24
O	1.46	0.59	0.74	0.50	0.46	1.11	0.42	0.64	0.45	0.61
Others	Balance									

**Table 4:** Elemental distributions in fusion line with different gas atmospheres (mass fraction, %).

Location	Atmospheres	Al	Si	Mn
I zone	Air	4.84	0.88	0.81
	Ar	5.7	0.88	1.08
II zone	Air	6.83	1.01	0.77
	Ar	9.37	1.24	0.69
III zone	Air	3.51	1.14	0.96
	Ar	4.28	0.95	0.97
